# Atomic Friction Processes of Two-Dimensional Materials

**DOI:** 10.1021/acs.langmuir.3c01546

**Published:** 2023-10-25

**Authors:** Yiming Song, Ernst Meyer

**Affiliations:** Department of Physics, University of Basel, Basel 4056, Switzerland

## Abstract

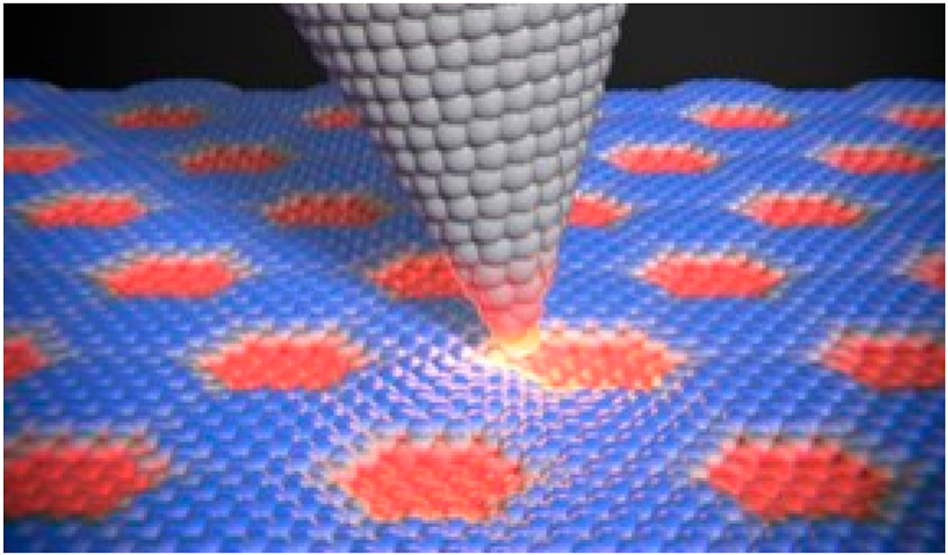

In this Perspective, we present the recent advances in atomic friction measured of two-dimensional materials obtained by friction force microscopy. Starting with the atomic-scale stick–slip behavior, a beautiful highly nonequilibrium process, we discuss the main factors that contribute to determine sliding friction between single asperity and a two-dimensional sheet including chemical identity of material, thickness, external load, sliding direction, velocity/temperature, and contact size. In particular, we focus on the latest progress of the more complex friction behavior of moiré systems involving 2D layered materials. The underlying mechanisms of these frictional characteristics observed during the sliding process by theoretical and computational studies are also discussed. Finally, a discussion and outlook on the perspective of this field are provided.

## I Introduction

Friction is a universal phenomenon existing in our daily life spanning length scales from earthquakes and mechanical bearings down to nanoscale motors.^1^ A fundamental understanding of origin of friction on the nanoscale is of essential importance to find solutions to reduce energy dissipation and achieve wearless motion in microscopic and nanoscopic electromechanical devices.^1,2^ With the advent of atomic force microscopy^3^ and its adaption^4,5^ to study friction of single asperities on the nanoscale, significant advances in detecting forces on the sub-nanonewton level resisting relative motion between probe and substrate have been witnessed in the past three decades. This leads to a profound understanding of the physical processes underlying friction at the atomistic level.

In friction force microscope experiments, a sharp tip is dragged via a spring along the crystalline surface at a constant normal load and sliding velocity. When the cantilever starts moving relatively to the substrate, the spring extends, which leads to an increase of lateral force and flattening the energy barrier for the tip to overcome. Instabilities are involved during sliding. Since the first atomic stick–slip process represented by Mate et al. using a tungsten tip sliding on graphite surface,^7^ to date an ever-growing number of atomic friction studies have been demonstrated on various surfaces and external conditions in the field of nanotribology. Atomic stick–slip instabilities, a fascinating highly nonequilibrium process, have been observed on numerous material surfaces including metals,^8−10^ semiconductors,^11^ and insulators.^12^ In FFM experiments, the effects of normal load, sliding velocity, temperature, and scanning direction on atomic friction have been investigated. The role of the normal load in atomic friction processes was studied by Socoliuc et al. in FFM measurements between a silicon tip and NaCl crystal surface.^13^ They observed a transition from dissipative stick–slip motion to smooth sliding which is reversible only depending on the applying load.^13^ This is a universal physical phenomenon confirmed by subsequent experiments on other surfaces such as graphite^14^ and gold.^9^ Furthermore, they developed a method to switch friction on and off by applying external mechanical excitations of the sliding system perpendicular to the contact plane.^15^ The velocity dependence of atomic friction between the AFM tip and crystal surface has been experimentally revealed by Gnecco et al.,^12^ representing a logarithmic dependence of friction force. Several measurements based on friction force microscope confirmed this typical logarithmic regime validate spanning many decades of velocity.^16−18^ The observed velocity dependence of friction can be well described under the frame of thermally activated Prandtl–Tomlinson (PTT) model^19,20^ based on reaction rate theory. The PTT model also successfully interpreted the relevant atomic friction measurements with varying temperatures showing an increase in friction with decreasing temperature.^21^ To investigate the origin of friction at the atomic scale, both noncontact and contact friction measurements^22,23^ are performed on superconductors across the critical temperature. Thus, the electronic and photonic contributions could be determined separately. In fact, the contact is not always a single atom as described by the PTT model but hundreds of atoms within the contacting region. Thus, a sharp tip and a blunt tip lead to different friction forces at the single cleavage step edge.^24^ The certain degree of commensurability between the tip and sample raises the issue of anisotropic orientational dependence of atomic friction.^25^ Another anisotropy effect relying on the sliding direction of AFM tip is also observed,^26,27^ which originated from the structural anisotropy of the sample surface. Further studies based on molecular dynamics were performed to obtain a better understanding of atomic friction process in the buried contacting interface.^28^

**Figure 1:**
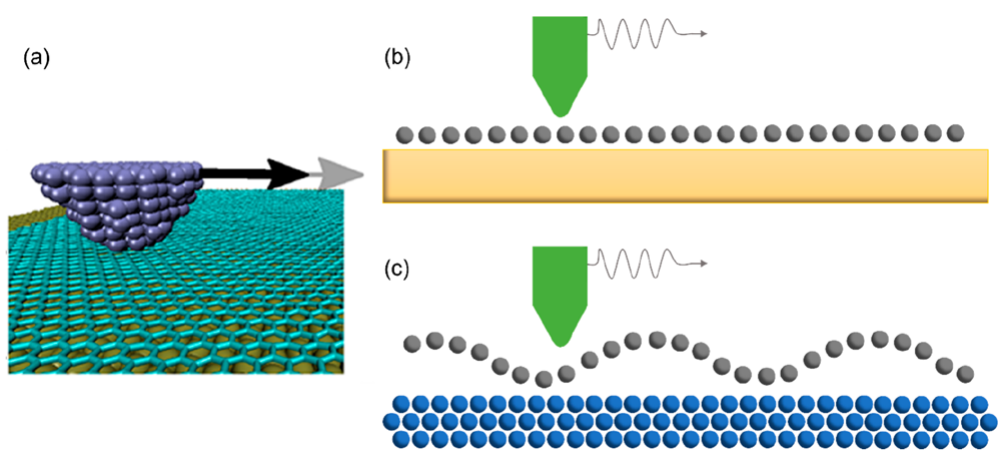
(a) Schematic of sliding friction between the AFM tip and the surface of 2D materials. Reproduced with permission from ref (6). (b) Atomic friction on flat 2D materials surface. (c) Sliding friction on corrugated moiré superstructure formed by a 2D layer and subsurface.

The above-mentioned examples are illustrated normally with ionic crystals or layered materials, which are widely used due to the easy preparation of atomically flat lattice planes. The role of crystallography in atomic friction is fundamental. Measurements between the AFM tip and many other surfaces with different chemical natures have been performed because the sample can be freely selected in a wide range. These studies led to the successful examination of friction processes at the atomic level. When the thickness of the sample goes down to monolayer, 2D materials emerge of which remarkable mechanical, electronic, and optical properties have been revealed experimentally.^29^ Its tribological performance is of both fundamental and practical importance for massive applications emerging in microscopic devices. The stacking order between the 2D layer and subsurface gives rise to the moiré superstructure,^30^ a novel degree of freedom to effectively modulate the frictional properties. In this Perspective, we focus on two aspects of atomic friction processes on 2D materials: atomically flat 2D surfaces and moiré corrugated superlattice induced by surface reconstruction (Figure 1).

## II Atomic Friction on Flat 2D Materials Surfaces

In recent years, growing interest was focused on the atomic-scale friction behavior between FFM probe and two-dimensional (2D) materials such as graphene, h-BN, and transition metal dichalcogenides (TMDs), where atomically flat nanocontact can be investigated. By way of comparison, such thin surfaces consisting of one or several layers of atoms on one hand exhibit excellent friction reduction properties^31^ akin to its bulk lubricants and on the other hand reveal a variety of unique tribological performances, such as layer dependence,^32^ friction anisotropy,^33^ and commensurability.^25^ Furthermore, the atomic stick–slip friction of 2D lamellar materials can be tuned over a wide range by chemical modifications,^34−36^ defect engineering,^37^ and external conditions.^38^

These layered materials are predicted to show ultralow friction and wearless motion when it is scratched by the AFM tip based on the fact of their high in-plane elastic modulus due to the intralayer covalent bonding, weak van der Waals interaction between probe and surface, and high chemical inertness. These unique features provide a new promising solution in solid lubrication by coating the substrate with 2D material layers.^39^ For such applications, the tribological properties of 2D material surfaces are of particular importance from scientific and technological perspectives. Several AFM measurements report the reduction of friction on graphene patches in contrast to a variety of surrounding substrate surfaces including metal,^31^ semiconductor,^40^ and insulator.^32^ More interestingly, friction on suspended graphene is investigated as well showing similar characteristics as on graphene supported by a rough substrate.^32^

**Figure 2:**
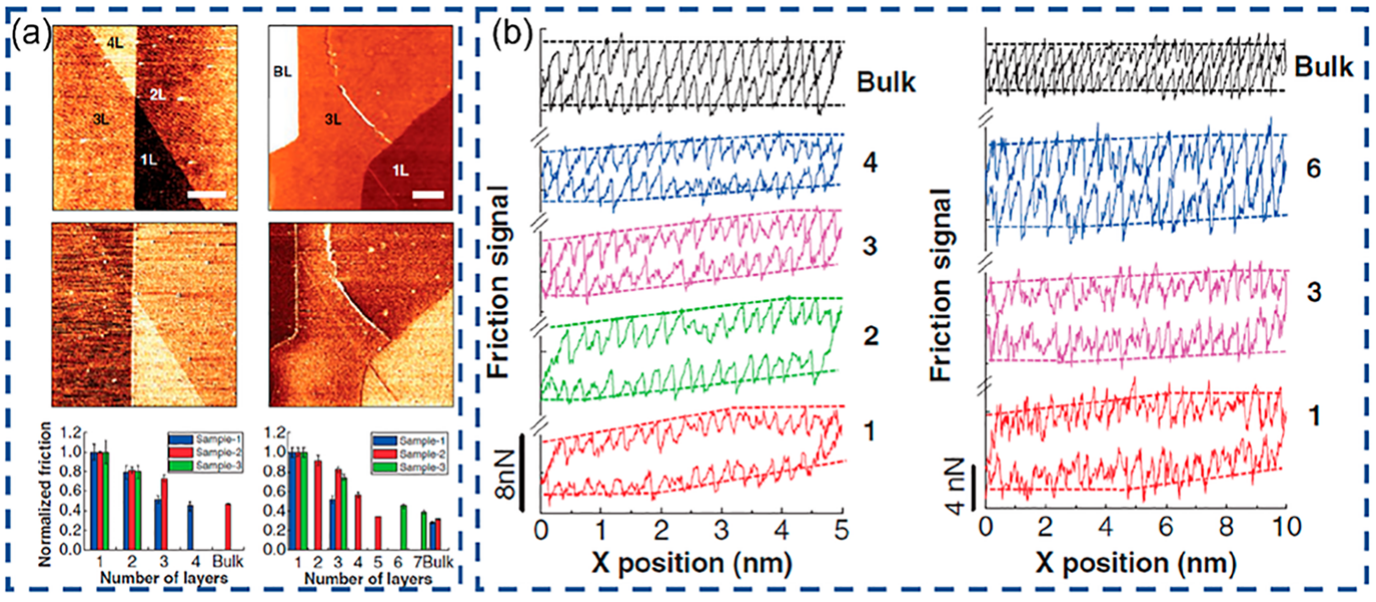
Thickness dependence of friction and corresponding atomic stick–slip friction loop detected on graphene and MoS_2_, respectively. Reproduced with permission from ref (32). Copyright 2010 AAAS.

Filleter et al. have demonstrated atomic friction measurements between single asperity and graphene epitaxially grown on SiC(0001) where a typical hexagonal stick–slip motion has been observed in both monolayer and bilayer graphene under ultrahigh vacuum (UHV) conditions.^41^ Single-layer graphene exhibits a higher average friction than bilayers. They suggested the difference in electron–phonon couplings for monolayer and bilayer graphene gives rise to the revealed friction contrast. The evolution of nanofriction from single layer to few layers has been studied by Lee et al. for a variety of mechanically exfoliated 2D materials under ambient conditions.^32^ They found a negative layer dependence on friction in all measured surfaces and distortion of the regular stick–slip motion. Out-of-plane deformation of 2D layered materials, also referred to as the puckering effect (as shown in Figure 2), around the tip accounts for the layer dependence resulted from weak interaction between layer and substrate and low out-of-plane bending stiffness. This finding is further confirmed by the measurement in the case of graphene on mica substrate where high adhesion in between resulted in lower friction.^32^ Recently, Rejhon et al. have investigated interfacial transverse shear modulus of epitaxial mono- and bilayer graphene on a substrate under ambient conditions, revealing that larger shear softness of a single atomic layer leads to larger amounts of energy dissipation.^42^

For mechanically exfoliated 2D material films, the anisotropy of friction has been reported in atomic friction experiments and simulations. An impressive example of friction anisotropy is given by graphene transferred onto a SiO_2_ substrate. Choi et al. observed significant anisotropic ratios of friction up to 215% with a periodicity of 180° on each frictional domain.^33^ This directional dependence on friction arises from the ripple structure resulting from interfacial inhomogeneous interaction during the sample preparation process. Another recent example of anisotropic friction is related to transition metal dichalcogenides (TMDs). Vazirisereshk et al. studied the atomic friction acting on single asperity sliding on wrinkle-free MoS_2_ surface by means of FFM and MD simulations.^43^ A 2-fold symmetry has been obtained in both experiments and simulations due to the distorted, low-symmetry tip–sample potential energy surface. Another factor that should be taken into account is the self-assembly of adsorption of contaminants on surfaces of 2D materials in ambient conditions leading to the frictional anisotropy in FFM measurements^44,45^ as well.

The dependence of friction on 2D materials on the external applied load is of fundamental scientific interest where nonlinear dependence of friction on normal load could be fitted with DMT or JKR relations.^2^ The load dependence of friction could be tuned by applying mechanical strain in the suspended single-layer graphene which is attributed to the change of contact quality between sliding AFM probe and surface.^38^ As discussed above, the contact region in realistic AFM experimental measurements was interpreted to be hundreds of atoms, where the degree of commensurability between the tip and substrate plays an important role in the atomic friction features. The combination of the quantity of atoms in contacting area and quality of contacting interface determines the frictional response of 2D materials surface such as strengthening effect.^46^ Actually, high contact pressure can lead to the intermittent formation of covalent bonds which limits the super low friction at tip–sample interface.^47^ As for the relative scanning speed, the friction of monolayer 2D layer as a function of sliding velocity shows a logarithmic dependence^48^ similar to the observed results from the bulk sample. Zhao et al. studied the dependence of friction on temperature observing the exponential increase with decreasing temperature in the case of MoS_2_.^11^

As discussed above, within the family of 2D layered materials, graphene, h-BN, and TMDs have shown similar frictional performance such as extremely low friction, layer dependence, and friction anisotropy; however, the fundamental understanding of the role of the chemical identity of these 2D materials surface remains unclear. To this end, in situ AFM measurements of monolayer graphene and MoS_2_ with the same probe have demonstrated that graphene exhibits a lower friction than MoS_2_ depending on the difference in energy barriers for the tip to overcome.^40^ In TMDs systems, lattice constants of the chalcogen also give rise to friction contrast, where the larger lattice constants lead to lower sliding friction.^49^ Furthermore, Zhou et al. found that the vertical interlayer force constant of various TMDs, changing transition metal, dominates the nanoscale friction behavior of MoS_2_ vs WS_2_ and MoSe_2_ vs WSe_2_.^50^

Chemical modification is a powerful tool to modulate the chemical composition of 2D layered materials surface, for example, hydrogenation,^35^ oxidation,^34^ and fluorination.^36^ Fessler et al. studied the atomic friction on pristine and hydrogenated graphene exfoliated on SiO_2_ substrate in a single measurement showing that the frictional behavior is the practically same on both surfaces once contamination adhering to hydrogenated regions is cleaned.^35^ However, a 6-fold enhancement of atomic friction measured on fluorinated graphene has been revealed by Kwon et al. under UHV conditions^36^ compared to the intrinsic layer. They attribute this observation to the enhancement of the out-of-plane bending stiffness of fluorinated graphene. A systematic study of friction on hydrogenated, fluorinated, and oxidized graphene, compared to pristine graphene, shows 2-, 6-, and 7-fold enhanced nanoscale friction on their surfaces, respectively.^34^ Relevant density-functional theory calculations indicate the main dissipation route of the out-of-plane vibrations. Corresponding functional group removal from 2D materials has been revealed as well. Felts et al. reported the bond scission technique induced by mechanical stress which enables the cleavage of chemical groups such as oxygen, fluorine, and hydrogen from graphene.^51^ Zambudio performed friction force measurement of atomic monovacancies on defective graphene prepared by means of Ar^+^ irradiation.^37^ A 5-fold enhancement of effective friction coefficient was observed with low density of atomic vacancies due to the chemical reactivity of dangling bonds and long-range strain distribution induced by the defect.

## III Sliding Friction on Moiré Superlattice

So far, we have discussed the atomic friction process on a 2D materials surface with atomic flatness. However, with the growing progress in the field of moiré materials, the influence of periodic long-range reconstruction induced by lattice mismatch between 2D layer and subsurface on the atomic friction process has opened a new nanofriction research frontier. In this section, we focus on the atomic friction behaviors on 2D materials with a moiré superlattice.

Variation of atomic friction due to superstructure was investigated experimentally and theoretically about one decade ago. Maier et al. experimentally studied the atomic friction process on reconstructed surface by depositing KBr film on NaCl(001) substrate for the first time,^52^ finding the modulation effects of friction force by long-range superstructure due to the lattice mismatch between these two materials. A tiny out-of-plane surface corrugation accounts for the modulation of the amplitude of the potential corrugation owing to the moiré superlattice. Filleter et al. used the AFM tip to slide graphene film grown on SiC(0001) with long-period superstructure.^53^ Although the lateral forces acting on the tip are modulated with reconstruction features, there is no variation in the energy dissipated during the sliding process. In the framework of the Tomlinson model, the influence of adsorbed molecules on friction was investigated by Tshiprut et al. by introducing local perturbations,^54^ and a second harmonic in the tip–surface interaction potential to mimic the disordered surface was taken into account by Fajardo and co-workers.^55^ After reviewing atomic friction measurements on superstructures, Steiner et al. extend the PT model to the ordered superlattice by modifying the substrate potential describing the interaction between the AFM tip and surface.^56^ They found two types of potential modulations for interpreting the corresponding observed atomic friction process:^56^ (i) amplitude modulation, multiplication of atomic potential by the superstructure potential; (ii) centerline modulation, superposition of atomic sinusoidal potential and long-period potential. These, in turn, give rise to the modulations of lateral force in both cases, as observed above. In contrast to amplitude modulation, the centerline modulation does not affect friction dissipation, as the trace and retrace of friction are consistently changed.^56^ The origin of the difference in these two types of modulations might be attributed to the unique physical and chemical properties of two-dimensional layered materials. The weak interfacial van der Waals interactions between tip and surface in combination with high chemical inertness strong intralayer covalent bonding of 2D materials may be the main reasons leading to the superposition of the short-range and moiré-level potentials. Another specific example of the surface reconstruction is the Au(111) herringbone which is imaged successfully by contact friction force microscopy under UHV conditions.^9,57^ As for noncontact friction, charge-density waves where a superstructure is formed by a charge redistribution make a key contribution to energy dissipation compared to metal phase in the case of NbSe_2_.^58^

These examples are the first observations of modulated lateral force induced by moiré superlattice at the atomic level. However, the origin of the modulation effect is not fully understood, as it could be attributed to geometrical effect or other factors such as internal degree of freedom of the supercell. In fact, the Tomlinson model developed above assumes AFM tip sliding over a rigid crystalline surface^1^ represented by fixed periodic potentials. Thus, the classic PT model fails to describe the real scenario during the sliding process considering the in-plane and out-of-plane deformations of the moiré supercell. This local straining in both directions induced by scanning probe was confirmed by STM measurement of graphene on an h-BN substrate.^59^ Furthermore, the effects of periodic dimensions and mechanical and electrical properties of moiré superlattice on the frictional properties of 2D material should not be ignored.

As indicated by Filleter et al.,^53^ the moiré superstructure involving 2D atomic layers offer a unique platform to gain fundamental understanding of friction on a variety of ordered superstructures, where the topographic, mechanical, and electrical properties could be tuned widely.^30^ Chan et al. measured friction vs the relative orientation of graphene film grown on Pt(111) surface in UHV.^61^ They observed that in addition to atomic stick–slip motion, lateral force modulation critically depends on the moiré pattern originating from the natural lattice misfit between the graphene film and Pt(111) surface. Similar lateral force modulation induced by the moiré supercell was revealed by means of molecular dynamics (MD) simulations which is attributed to the geometric corrugation of graphene.^62^ Long-range stick–slip dynamics was observed by Shi et al. in the case of graphene on Ru(0001) depending on the interfacial interaction of the heterogeneous structure.^63^ Zheng et al. represent similar experimental observations of graphene on Ge(111) such as long-range centerline modulation of lateral force along the moiré pattern.^64^ Furthermore, they proved that this nanoscale confinement induced by moiré superlattice leads to a strong suppression of out-of-plane of the 2D atomic layer even when the surface was chemically modified by fluorination or oxidation where normally significant enhancement of friction emerges. Because of a similar reason, graphene confined with the moiré pattern on Pt(111) substrate gives rise to substantial enhancement of the load carrying capacity during tip sliding.^39^ Further study shows the capability to tune the moiré level lateral force by applying external bias voltage introducing current transfer from the conductive AFM probe to graphene on Ru(0001).^65^

**Figure 3:**
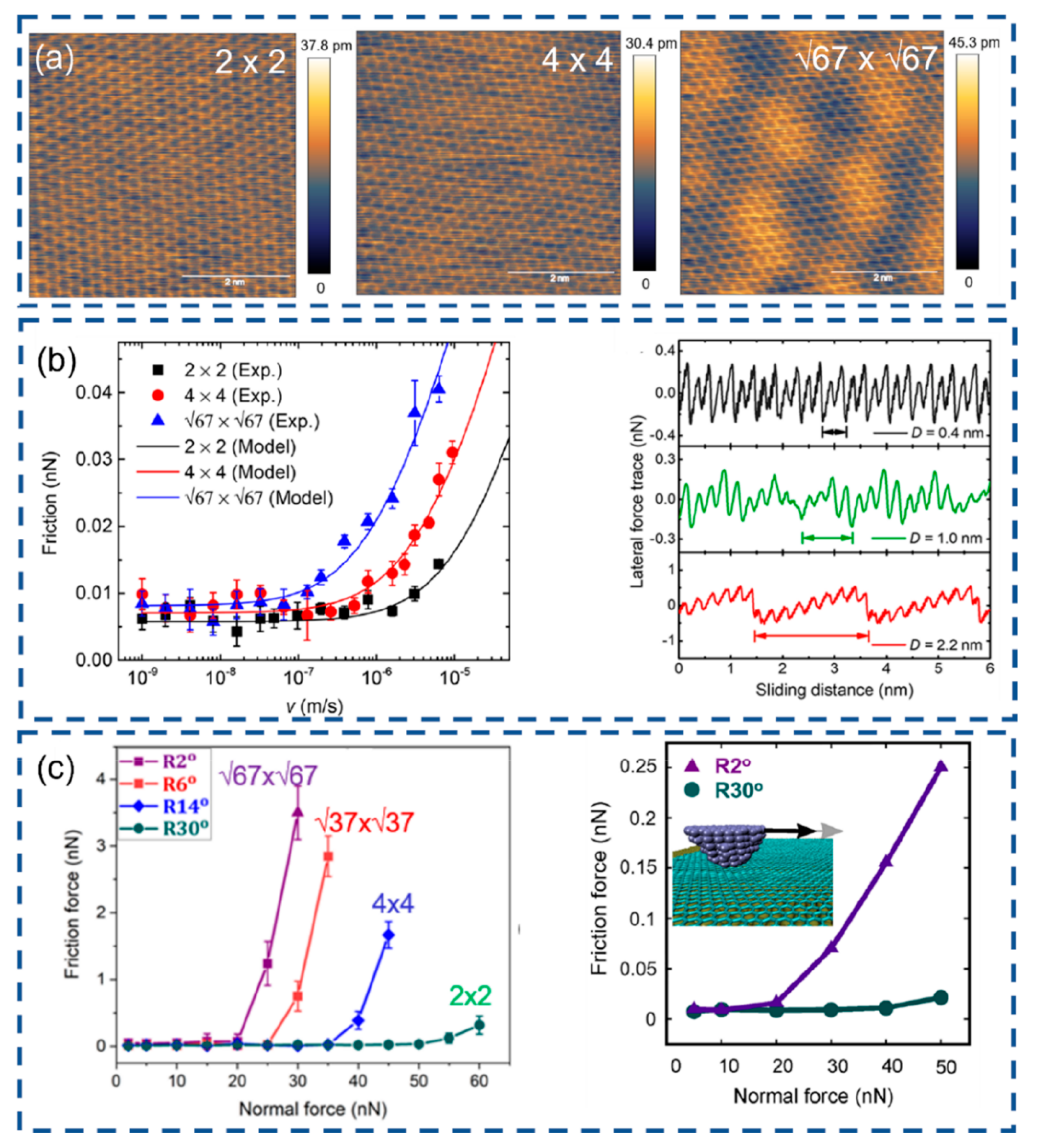
(a) Topography images of the moiré superlattice formed by graphene and Pt(111) surface. (b) Velocity dependence of the moiré friction. (c) Load dependence of the moiré friction. Reproduced with permission from ref (6).

It is of great interest and importance in both technological applications and scientific research to find the fundamental mechanism of moiré friction in addition to atomic stick–slip motions. To this end, Song et al. conducted the friction measurements on superlattice of graphene-coated Pt(111) surfaces (Figure 3a) with sliding velocities under UHV conditions.^60^ Two distinct regimes of friction with sliding velocities have been revealed: (i) friction force remains ultralow and nearly constant below some threshold; (ii) at high velocities, the logarithmic frictional dependence on speed appears, as shown in Figure 3b. In addition, the interfacial twist angle leading to different periodicities of moiré superstructures plays an important role in the threshold velocity separating the two frictional regimes where the larger superlattice dimension results in lower transition velocity. Based on the measurements and simulation results, it was proven that this abnormal speed dependence of friction is due to a new dominant energy dissipation route, moiré level elastic deformation, and relaxation occurring at the ridge. This is in line with the previous theoretical study conducted by Anderson et al. which emphasized the importance of elastic deformation in the atomic friction process on superstructures.^66^ To bridge the gap of sliding velocities between MD simulations and experiments, a phenomenological model is derived, showing the ability to calculate the relevant friction force in excellent agreement with experimental results.

In addition to the velocity dependence, the load dependence of atomic friction was also studied with the same graphene/Pt(111) system. Figure 3c demonstrates a transition from superlubric sliding to a highly dissipative shear process with increasing normal loads.^6^ This transition is influenced by moiré size as well, showing the capability of tuning frictional dissipation widely in a simple and reversible manner. These findings could be explained very well in the framework of the dissipative moiré-scale stick–slip mechanism. Further studies that investigated the atomic friction on graphene/h-BN superlattices with larger periodicities^67^ confirm that moiré-level stick slip behaviors attributed to the in-plane elastic deformation and release of the graphene superstructure exist up to the moiré pattern with a period of ∼15 nm.^67^ Based on MD simulations, Huang et al. reveal the underlying mechanism that moiré level stick–slip originated from the coupling between in-plane strain and out-of-plane deformation of the graphene layer on an h-BN substrate.^68^

**Figure 4:**
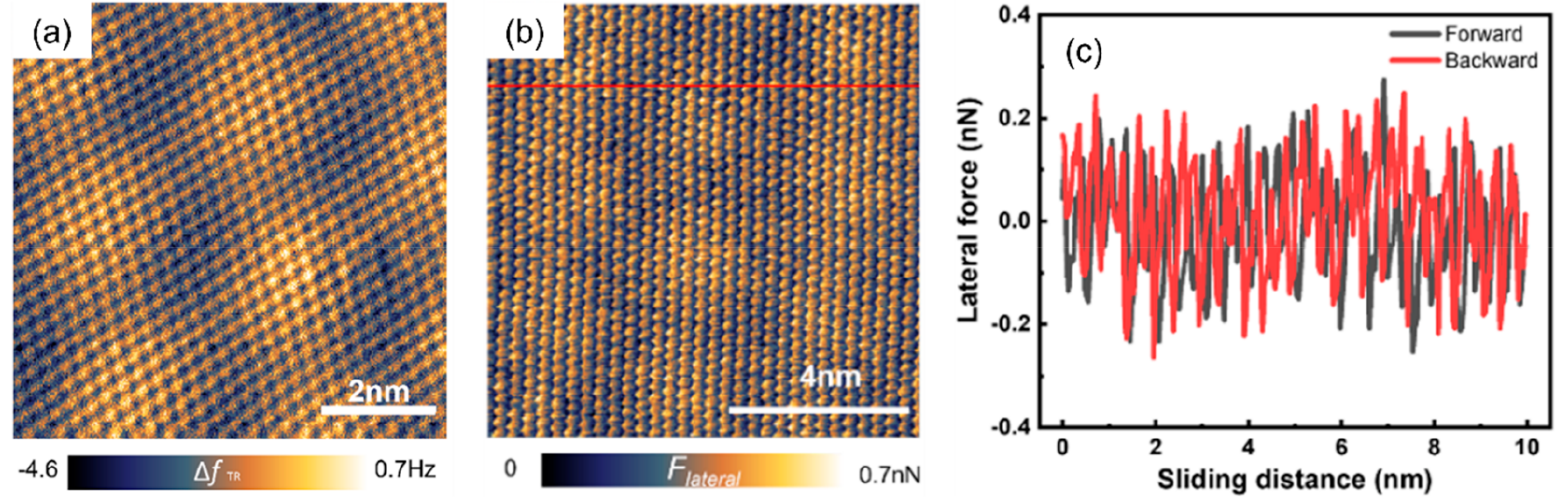
(a, b) High-resolution torsional frequency shift and lateral force images of the moiré superlattice formed by MoS_2_ and Au(111) surface.^70^ (c) Centerline modulated friction loop measured on the MoS_2_/Au(111) moiré system. Reproduced with permission from ref (70). Copyright 2022 ScienceDirect.

All the cases discussed above are related to the moiré systems with relatively weak van der Waals interlayer interaction, such as for the case of graphene/h-BN heterostructure microscale robust superlubric sliding has been uncovered between these two surfaces.^69^ The influence of the strength of interfacial interactions on moiré friction should be investigated. Song et al. prepared MoS_2_ film on Au(111) surface under UHV conditions^70^ to exclude the contaminants.^45^ Relatively strong adhesion has been predicted by previous DFT calculations for the MoS_2_/Au(111) system.^71^ It was confirmed by another FFM experiment revealing pronounced adhesion and higher sliding friction between MoS_2_ and the Au-coated tip, in contrast to the silicon tip.^72^ The friction force measurements of the MoS_2_/Au(111) system were carried out, revealing apparent independence of friction on load with a relatively large moiré size of 3.3 nm,^70^ in contrast to the previously reported moiré level friction at the ridge leading to a highly dissipated regime. The puckering effect is not observable when analyzing the corresponding lateral force trace and retrace (Figure 4). It is attributed to the relative rigidity of the moiré superlattice originating from a natural misfit between MoS_2_ and the gold surface. The disappearance of the “strengthening” effect (Figure 4c) confirms the strong suppression of both local in-plane and out-of-plane deformation of the MoS_2_ layer scanned by an AFM probe.^70^ The preparation and friction measurement of the MoS_2_ surface under UHV conditions are important for the experimental findings to exclude potential contaminations. This paves the way to control moiré friction by varying the strength of interaction of 2D atomic layer with the underlying substrate.

## IV Conclusion

Over the course of this Perspective, we have emphasized the variety of atomic friction processes on 2D materials surfaces which has been investigated by friction force microscopy. Material identity, thickness, sliding direction, load, velocity, and chemical modifications have significant effects on frictional behaviors of 2D layers. More importantly, we have represented that moiré superstructures involving a 2D atomic layer provide a new dominant energy dissipation route in addition to atomic stick–slip dynamics. We want to emphasize the important role of the elastic deformations, which extend over a larger length scale related to the moiré pattern. Because of the large extension of the elastic deformations during the stick phase, a larger energy can be dissipated in the slip phase, which explains the dominance of moiré friction compared to atomic friction. This behavior is observed not only experimentally but also by MD simulations and is well represented by the phenomenological two-state model. Despite being very successful in atomic friction studies on 2D materials, there is still much work to be done. Here we emphasize a few directions that we feel are important and need to be further addressed:

- Determination of the friction dissipation of unideal 2D layered materials surface with various types of defects such as zero-dimensional point defects and line defects. More experiments, particularly in UHV, are needed to reveal the mechanism in the beginning. Subsequent systematic studies on defect friction in various environments could lead to further understanding of the corresponding atomic frictional behaviors for more realistic application scenarios.
- As discussed above, the moiré superstructure gives rise to various frictional characteristics due to its unique mechanical properties. Phase transition of 2D layered materials provides another new degree of freedom for varying not only electronic properties but also frictional response of atomic surfaces. Further AFM studies on corrugated moiré surfaces in combination with phase transition would be useful to investigate atomic-scale and moiré-scale frictional behaviors of 2D material over phase transitions.
